# Whole body irradiation combined with adoptive T cell transfer results in regression of high-grade intraepithelial neoplasia and long-term persistence of tumor-specific CD8+ T cells in TRAMP mice

**DOI:** 10.1186/2051-1426-1-S1-P37

**Published:** 2013-11-07

**Authors:** Lindsay K Ward-Kavanagh, Timothy K Cooper, Todd D Schell

**Affiliations:** 1 Microbiology and Immunology, Pennsylvania State Univ. Col. of Med., Hershey, PA, USA; 2 Comparative Medicine, Pennsylvania State Univ. Col. of Med., Hershey, PA, USA

## 

Lymphodepleting whole body irradiation (WBI) can enhance adoptive T cell transfer-mediated tumor regression in a variety of experimental models and a subset of patients. We investigated the potential of this approach to impact established prostate cancer using the clinically relevant and immunosuppressive TRAMP model of prostatic adenocarcinoma. 18-week-old TRAMP mice bearing high-grade prostatic intraepithelial neoplasia (PIN) were treated with 475cGy WBI followed by adoptive transfer of naïve T cell receptor transgenic CD8+ T cells (TCR-IV) specific for the Site IV determinant in SV40 T-antigen. TCR-IV cells proliferated in both irradiated and non-irradiated TRAMP mice, but accumulated to significantly higher levels and persisted for 21 days in irradiated mice. Neither treatment group developed a population of IFNγ-producing TCR-IV cells in the prostate, but a significantly larger number of Granzyme B-producing cells infiltrated the prostates of mice conditioned with WBI. The addition of WBI also delayed the onset of tolerance, as the TCR-IV cells in irradiated mice remained responsive to immunization. WBI alone and WBI with adoptive transfer tended to reduce the size of the urogenital tract and reduce the incidence of adenocarcinoma at 28 weeks of age, but no regressions were observed. However, a subset of TRAMP mice treated with two rounds of WBI-enhanced adoptive immunotherapy exhibited only low- or moderate-grade PIN at 28 weeks of age, indicative of tumor regression. The remainder of mice in this group exhibited no disease progression beyond high-grade PIN, and all mice in the group maintained a detectable population of TCR-IV cells 49 days after the second adoptive transfer. In contrast, at least a subset of mice in remaining groups progressed to adenocarcinoma, and all lacked detectable TCR-IV cells. These results demonstrate that WBI conditioning extends the persistence of effector phenotype tumor-specific T cells in the highly tolerogenic microenvironment of the tumor-bearing TRAMP prostate, and suggest that multiple rounds of therapy can promote regression of established prostate lesions.

